# Predictors for health facility delivery in Busia district of Uganda: a cross sectional study

**DOI:** 10.1186/1471-2393-12-132

**Published:** 2012-11-20

**Authors:** Agnes Anyait, David Mukanga, George Bwire Oundo, Fred Nuwaha

**Affiliations:** 1 Makerere University School of Public Health, P.O Box 7072, Kampala, Uganda; 2 Busia district, Directorate of Health Services, P.O. Box 124, Busia, Uganda

**Keywords:** Home deliveries, Male involvement, Birth plan, Sub-Saharan Africa

## Abstract

**Background:**

Among the factors contributing to the high maternal morbidity and mortality in Uganda is the high proportion of pregnant women who do not deliver under supervision in health facilities. This study aimed to identify the independent predictors of health facility delivery in Busia a rural district in Uganda with a view of suggesting measures for remedial action.

**Methods:**

In a cross sectional survey, 500 women who had a delivery in the past two years (from November 16 2005 to November 15 2007) were interviewed regarding place of delivery, demographic characteristics, reproductive history, attendance for antenatal care, accessibility of health services, preferred delivery positions, preference for disposal of placenta and mother’s autonomy in decision making. In addition the household socio economic status was assessed. The independent predictors of health facility delivery were identified by comparing women who delivered in health facilities to those who did not, using bivariate and binary logistic regression analysis.

**Results:**

Eight independent predictors that favoured delivery in a health facility include: being of high socio-economic status (adjusted odds ratio [AOR] 2.8 95% Confidence interval [95% CI]1.2–6.3), previous difficult delivery (AOR 4.2, 95% CI 3.0–8.0), parity less than four (AOR 2.9, 95% CI 1.6–5.6), preference of supine position for second stage of labour (AOR 5.9, 95% CI 3.5–11.1) preferring health workers to dispose the placenta (AOR 12.1, 95% CI 4.3–34.1), not having difficulty with transport (AOR 2.0, 95% CI 1.2–3.5), being autonomous in decision to attend antenatal care (AOR 1.9, 95% CI 1.1–3.4) and depending on other people (e.g. spouse) in making a decision of where to deliver from (AOR 2.4, 95% CI 1.4–4.6). A model with these 8 variables had an overall correct classification of 81.4% (chi square = 230.3, P < 0.001).

**Conclusions:**

These data suggest that in order to increase health facility deliveries there is need for reaching women of low social economic status and of higher parity with suitable interventions aimed at reducing barriers that make women less likely to deliver in health units such as ensuring availability of transport and involving spouses in the birth plan.

## Background

Early and regular attendance of antenatal care (ANC) and delivery under supervision of a skilled attendant (defined as a midwife, nurse trained as midwife, or a doctor) is associated with improved outcome regarding maternal and peri-natal health as well as decrease in maternal and peri-natal deaths [1-4]. Because professional health care during childbirth is pivotal for child and maternal health, it is included as one of the process indicators to assess progress towards the Millennium Development Goal Number 5 of improving maternal health [5]. Contrasted to high income countries, in the low income countries of sub-Saharan Africa safe delivery with skilled supervision can only take place in health facilities as provision of domiciliary delivery is not feasible [6,7]. However, in the low income countries of sub-Saharan Africa, attendance of ANC falls short of WHO recommendations with most mothers initiating ANC after the first semester and attending for less than the recommended four visits. The proportion of women delivering in health facilities under supervision of a skilled attendant is also alarmingly low [8]. In Uganda for example, almost all (94%) of the pregnant women receive some antenatal care from a skilled provider but with only 47% of pregnant women in the country receive at least four antenatal visits and a mere 17% receiving their first antennal visit during the first trimester [9]. Furthermore, only 41% all the births occur within health facilities. The situation is more serious in rural areas where only 36% of the deliveries occur in health facilities compared to 79% for urban areas.

Previous studies have identified factors predicting the delivery care to include cultural, socioeconomic, demographic and service accessibility factors with low maternal or paternal educational attainment, low socioeconomic status, rural residence, young maternal age, and high-order births have being observed to be associated with high probabilities of deliveries outside a health facility [10,11]. It is important, however, to understand the specific factors that are important in various settings, since these may vary considerably [12,13]. Understanding predictors of health facility deliveries is a prerequisite for suggesting remedial measures.

## Methods

### Study area

The study was carried out in Busia district, Eastern Uganda near the border with Kenya. Busia district has one county, 10 sub-counties, one town council, 58 parishes, 474 villages and about 45,500 homesteads. The District has a population of 251,571 people spread over a land area of 743 km^2^. About 90% of the population resides in rural areas. Ten health centres offer maternity services. About 60% of the population is within five kilometres of a health centre offering maternity services. It is estimated that about 20% of all pregnant women deliver in health facilities. The district has a total road network of 710 km composed of 30 km of tarmac roads and 56 km earth gravel. Community roads cover about 200 km, while feeder road coverage is 251.3 km or 62% of the district [14].

### Study population

The study population was women who had A sample size of 500 had at least 80% power of detecting differences of 15% between the proportions of women delivering in health units and at home based on the Uganda demographic and health survey of 2006 [9]. a delivery within the last two years (from November 16 2005 to November 15 2007) and resided within five kilometres of a health facility providing delivery services. Five hundred women were selected from 25 villages. The villages were selected randomly from a listing of 200 villages using a table of random numbers. Starting from a random direction in the middle of the village, households were visited in sequence until when the required number of 25 women was obtained. Where more than one woman was found in the household one was randomly selected. Of the 505 eligible women identified for the interviews only 5 (<1%) refused to participate citing lack of time as the reason for refusal.

### Data collection

In this cross sectional study, data collection started from 15 November 2007 and lasted for 30 days. Trained research assistants supervised by one of the principal investigators (AA) interviewed the women regarding date of most recent delivery, mother’s age at time of delivery, occupation of respondent and of spouse, education status of respondent and of spouse, marital status, history of last pregnancy and delivery, attendance of antenatal care, timing of antenatal care, number of times of attendance of antenatal care, presence of a birth plan, time from onset of labour to delivery, place of delivery, knowledge of expected date of delivery, parity, previous delivery places, previous delivery outcomes for both mother and babies, distance to health facility, affordability of delivery services, availability of transport, availability of drugs, health personnel, equipment and supplies, perceptions of health facility delivery, attitude of health workers, preferred delivery positions, preference for disposal of placenta and mother’s autonomy in control of finances and in decision making.

In addition the household socio economic status was assessed according to the method described in the Uganda demographic and health survey based on type of housing and assets owned [9]. All households were categorized by 5 quintiles and then regrouped into two with low socio-economic status (SES) defined as an SES in the bottom 3 quintiles of the wealth index and a high SES was defined as those in fourth and highest quintiles.

### Analysis

The outcome variable was place of delivery for the most recent pregnancy. Differences in proportions between facility and non-facility deliveries were compared using the Yates corrected Chi-square test (χ^2^) or Fisher’s exact test when appropriate, and differences in means were compared using the Student’s *t*-test. A two-sided *P*-value < 0.05 was considered statistically significant. Crude Odds ratios (COR) with 95% confidence intervals (CI) were calculated after bivariate analysis. Logistic regression was used to assess the effect of various factors on place of delivery. All those factors that were found to be significantly associated with delivery at a health facility from bivariate analysis were considered for logistic regression analysis to generate a model to explain delivery at a health facility. Using backward likelihood ratio method, logistic regression analysis was run and a test of the full model with the predictors was assessed for the percent of variance it explains. The statistical software *STATA* (Stata Corporation, College Station, TX) version 9 was used to analyze the data. Standard *STATA* commands were used for adjusting the data for clustering at the village level. The variables that were significant at bivariate analysis were assessed for multi-co linearity before the logistic regression. Where two variables were highly correlated (P < 0.01) only one variable was included in the model. The basis for inclusion was which of the variables appear logically essential to the model.

### Ethical considerations

Ethical clearance to carry out the study was obtained from Makerere University School of Public Health institutional review board and the Uganda national council for science and technology. All women who participated gave verbal consent to the interviews.

## Results

### Place of delivery

The results showed that out of 500 women interviewed, 227 (45.4%) delivered in health facilities and 288 (58%) delivered within the last 12 months. Of the 227 women who delivered at a health facility, 159 (70%) deliveries were in a public health facility, while 68 (30%) were in a private health facility. Of the 273 deliveries outside a health facility, 249 (91.2%) were at the respondent’s home, 20 (7.3%) at a home of a traditional birth attendant (TBA), and four (1.5%) on the way to a health facility. A variation was observed between actual place of delivery and a woman’s initial planned place of delivery. From the interviews, 400 out of the 500 (80%) respondents said they had planned to deliver at a health facility, 89 out of 500 (17.8%) had planned to deliver at their own home while eight (1.6%) admitted to have planned to deliver with a TBA. Only three out of 500 (0.6%) said they had not planned a place of delivery.

### Demographic and socio-economic characteristics

About 23% of the respondent women were less than 20 years of age. The mean age of the women was 26 years with a standard deviation of seven years. The index pregnancy was the first pregnancy for 14% of respondent women. The mean number of gravidity was 4.2 (range 1–15). Women who had more than one pregnancy were asked if they ever had any adverse reproductive history. Nearly 10% of women reported having had a stillbirth, and 28% reported previous complicated or prolonged delivery. A majority of all the respondents were married (88.8%), and had attained primary level of education (65%). Most of the respondents were not employed for cash as their main occupation is peasant farming (86%). Almost all (95%) attended ANC at least once but only 13% booked in the first trimester although 60% attended for at least four times. About 16% of the women lived within one kilometre of a maternity facility, 51% within two kilometres and 79% within three kilometres. The majority of the women (56%) were of low social economic status.

Table 1 shows the associations between demographic and the socio-economic characteristics of respondents and place of delivery. Delivery in a health facility was more likely from mothers with high social economic status, if the mother was less than 20 years of age, if mother had at least secondary education, if husband had at least secondary education and if husband was employed for cash. Religion of mother, employment status of the mother time since last delivery and marital status of the mother did not influence place of delivery.

**Table 1:** Relationship between demographic and socio-economic characteristics and the place of delivery

**Characteristic**	**Health facility n (%)**	**Non health facility n (%)**	**Crude odds ratio (95% CI)**
**Age of mother at birth of last child**			
< 20 years	70 (62)	43 (38)	2.4 (1.5–3.8)
20 years and above	157 (41)	230 (59)	1.0
**Time since last delivery**			
1–12 months	153 (53)	135 (47)	0.87 (0.6–1.3)
13–24 months	120 (57)	92 (43)	1.0
**Religion of mother**			
Moslem	26 (46)	30 (54)	1.05 (0.6–1.9)
Christian	201 (45)	243 (55)	1.0
**Education status of mother**			
Secondary and above	60 (60)	40 (40)	2.1 (1.3–3.4)
None/Primary	167 (42)	233 (52)	1.0
**Occupation status of mother**			
Employed for cash	37 (57)	28 (43)	1.70 (0.98–2.98)
Employed not for cash	190 (44)	245 (56)	1.0
**Marital Status of mother**			
Married	211 (46)	244 (54)	1.57 (0.80–3.11)
Not married	16 (36)	29 (64)	1.0
**Education status of husband**			
No Husband	10 (22)	35 (78)	0.43 (0.20–0.94)
Secondary and above	117 (54)	100 (46)	1.92 (1.09–2.36)
None/ primary	100 (42)	138 (58)	1.0
**Occupation status of husband**			
No Husband	10 (22)	35 (78)	0.6 (0.3–4.5)
Employed for Cash	119 (64)	68 (36)	3.0 (2.0–4.5)
Employed not for cash	98 (37)	170 (63)	1.0
**House hold socio-economic status**			
High	42 (66)	22 (34)	2.6 (1.5–4.7)
Low/Medium	185 (42)	251 (58)	1.0

### Antenatal care attendance

Respondents were assessed for antenatal care attendance (ANC) in the most recent pregnancy including age of the gestation at first attendance of ANC, frequency of attendance of ANC, knowledge of the expected date of delivery (EDD) and presence of a birth plan. Also assessed was information given to mothers during ANC regarding the state of the baby in the womb and the mother’s condition during pregnancy. Table 2 summarizes the findings. Attending for ANC, for at least 4 times, being told that the baby was abnormal and having had a birth plan improved the chance of delivering in a health facility. Being told the condition of the mother, knowing expected date of delivery and timing of attendance for first ANC did not influence place of delivery.

**Table 2:** Relationship between antenatal care attendance and place of delivery

**Characteristic**	**Health facility n(%)**	**Non health facility n(%)**	**Crude odds ratio (95% CI)**
**Attendance of antenatal**			
Yes	225 (47)	251 (53)	9.9 (2.4–87.2)
No	2 (8)	22 (92)	1.0
Timing of first antenatal visit*			
7–9 months	29 (38)	48 (62)	0. (0.2–0.9)
4–6 months	158 (48)	176 (52)	0.6 (0.4–1.1)
1–3 months	38 (59)	27 (41)	1.0
Number of antenatal visits*			
1–3	65 (37)	110 (63)	1.9 (1.3–2.9)
> 3	160 (53)	141 (47)	1.0
Condition of baby during antenatal*			
Was not told	103 (48)	110 (52)	3.4 (1.2–9.9)
Normal	106 (44)	135 (56)	3.4 (1.2–10.9)
Abnormal	16 (73)	6 (27)	1.0
Condition of mother during antenatal*			
Was not told	115 (50)	113 (50)	1.4 (0.6–3.2)
Normal	98 (47)	122 (53)	0.9 (0.4–2.2)
Abnormal	12 (43)	16 (57)	1.0
Birth Plan*			
Present	144 (54)	122 (46)	1.9 (1.3–2.8)
Absent	81 (39)	129 (61)	1.0
Knew expected date of delivery*			
No	161 (45)	194 (55)	0.7 (0.5–1.1)
Yes	64 (53)	57 (47)	1.0

* Excludes those who did not attend antenatal.

### Previous delivery experiences

Association between the mother’s parity, previous pregnancy outcomes and place of delivery of the most recent child was assessed and the results shown in Table 3. The previous pregnancy outcome refers to the birth preceding the most recent one. Delivery in a health facility was likely if the mother had less than four births; delivery for past immediate preceding pregnancy was in a health facility and mother reported a complication in past delivery. Having caesarean delivery and time of onset of labour did not influence place of delivery.

**Table 3:** Relationship between parity, previous complications during delivery, time of onset of labour and place of delivery

**Characteristic**	**Health facility n(%)**	**Non health Facility n(%)**	**Crude odds ratio (95% CI)**
**Number of births**			
Less than four	141 (61)	91 (39)	3.33 (2.22–4.76)
Four and above	86 (32)	182 (68)	1.0
**Place of immediate past delivery**			
No past delivery	44 (63)	26 (37)	11.79 (6.12–22.89)
Health facility	152 (83)	31 (17)	34.16 (19.29–60.98)
Home	31 (13)	216 (87)	1.0
**Type of immediate past delivery**			
No past delivery	44 (63)	26 (37)	2.36 (1.36–412)
Caesarean	13 (57)	10 (43)	1.81 (0.72–4.57)
Vaginal	170 (41)	237 (59)	1.0
**Any complication during immediate past delivery**			
No past delivery	44 (63)	26 (37)	2.82 (1.61–4.96)
Yes	47 (70)	20 (30)	3.92 (2.16–7.18)
No Complication	136 (37)	227 (63)	1.0
**Time of onset of labour**			
During the day	159 (48)	175 (52)	1.31 (0.88–1.94)
During the night	68 (41)	98 (59)	1.0

### Health system factors

The effect of accessibility to the maternity facility and mother’s perception of the services offered in influencing place of delivery are shown in Table 4. Living within three kilometres of a health facility, having had access to transport and perceiving the cost of care at maternity service to be affordable increased the likelihood of health facility delivery. Time taken to reach maternity facility, adequacy of health workers or drugs and perceiving the facility to have had adequate privacy did not influence the place of delivery.

**Table 4:** Association between place of delivery and health system factors

**Characteristic**	**Health facility n(%)**	**Non health Facility n(%)**	**Crude odds ratio (95% CI)**
**Distance to nearest health facility with facilities for delivery**			
0–3 Kilometres	192 (49)	202 (51)	1.9 (1.2–3.1)
>3 Kilometres	35 (33)	71 (67)	1.0
**Time taken to nearest health unit with facilities for delivery**			
> 2 hours	42 (41)	61 (59)	0.8 (0.5–1.3)
0–2 hours	185 (47)	212 (53)	1.0
**Had access to transport (money or transport means at home at the time of time of delivery)**			
Yes	126 (37)	214 (63)	2.9 (1.9–4.4)
No	101 (63)	59 (37)	1.0
**The nearest maternity facility has adequate supply of drugs**			
Disagrees	127 (43)	168 (57)	0.8 (0.6–1.2)
Agrees	100 (49)	105 (51)	1.0
**The nearest maternity facility has adequate number of health workers**			
Disagrees	124 (43)	167 (57)	0.8 (0.5–1.1)
Agrees	103 (49)	106 (51)	1.0
**Satisfaction with the privacy at the nearest maternity facility**			
Not satisfied	139 (45)	167 (55)	1.0 (0.7–1.5)
Satisfied	88 (45)	106 (55)	1.0
**The cost of delivery at the nearest maternity facility is**			
Free	51 (41)	74 (59)	0.9 (0.6–1.4)
Affordable	56 (57)	43 (43)	1.7 (1.0–2.8)
Not affordable	120 (44)	156 (56)	1.0

### Beliefs associated with place of delivery

Health facility delivery was likely if mother agreed that health workers are kind, mother agreed that only women with problems during delivery need to deliver in health facilities, mother said that decision to attend ANC was made by herself, mother said that decision to deliver in health facilities was made by others, mother preferred lying on the back (supine) as delivery position and if the mother preferred health workers to dispose off the placenta. On the other hand, saying that health workers are corrupt, ability of maternity facility to do caesarean section and mother being in control of financial resources did not appear to influence place of delivery (see Table 5).

**Table 5:** Relationship between beliefs and place of delivery

**Characteristic**	**Health facility n(%)**	**Non health facility n(%)**	**Crude odds ratio (95% CI)**
**Health workers at the nearest maternity health facility are kind**			
Disagrees	121 (39)	186 (61)	0.5 (0.4–0.8)
Agrees	106 (55)	87 (45)	1.0
**Health workers at the nearest maternity health facility are corrupt**			
Agree	156 (44)	198 (56)	0.8 (0.6–1.3)
Disagree	71 (49)	75 (51)	1.0
**Its useless to deliver in a maternity facility where Caesarean Section can not be done**			
Agrees	107 (43)	142 (57)	0.8 (0.6–1.2)
Disagree	120 (48)	131 (52)	1.0
**Only women who get problems with delivery should deliver in health facilities**			
Agrees	14 (25)	43(75)	2.8 (1.5–5.6)
Disagrees	213 (48)	230 (52)	1.0
**The decision to seek antenatal care is generally made by**			
My self	166 (49)	174 (51)	1.6 (1.0–2.3)
Others *	61 (38)	99 (62)	1.0
**The decision regarding place of delivery is generally made by**			
Others *	78 (55)	65 (45)	1.68 (1.1–2.5)
My self	149 (42)	208 (52)	1.0
**Financial resources for health care are generally controlled by**			
Others **	221 (46)	255 (54)	2.6 (1.1–2.5)
My self	6 (25)	18 (75)	1.0
**My preferred position for delivery is**			
Lying on the back	198 (67)	96 (33)	11.1 (6.9–17.7)
Other positions ***	33 (16)	177 (84)	1.0
**The preferred person for disposal of placenta is**			
Health worker	183 (72)	71	11.8 (7.6–18.6)
Others ****	44 (21)	202	1.0

* Spouse, relative, friend, traditional birth attendant, or health worker.

** Spouse, relatives, *** Kneeling, Squatting, Sitting and lying on the side.

**** Relative, friend, traditional birth attendant.

### Independent predictors of health facility delivery

The independent factors influencing delivery in health facilities with their Adjusted Odds Ratios (AOR) are shown in Table 6. The model containing these eight predictor variables (being of high socio-economic status, having had a previous difficult delivery, parity less than four, preference of supine position for second stage of labour, trusting health workers in disposal of placenta, not having had difficulty with transport, being autonomous in decision to attend for antenatal care and depending on other people in making a decision of where to deliver from) could explain 62% of the observed variance and achieved an overall correct classification of the sample of 81.4%. The model was highly sensitive and specific. Among those who delivered in a health facility 182 out 227 (80.2%) were correctly classified, whereas among those who did not deliver in a health facility 225 out of 273 (82.4%) were correctly classified. This classification was significantly different from that observed by chance (model χ^2^ = 230.3, 2 log likelihood 810.7, degrees of freedom 8, P < 0.001).

**Table 6:** Independent predictors of health facility delivery with their Adjusted Odds ratios and 95% confidence intervals

**Variable**	**Adjusted odds ratio (95% CI)**
Household economic status (High)	2.8 (1.2–6.3)
Had access to transport (money or transport means at home during time of delivery)	2.0 (1.2–3.5)
The preferred person for placental disposal of placenta is health worker	12.1 (4.3–34.1)
The decision regarding place of delivery is generally not made by myself	2.4 (1.4–4.6)
Parity of mother is less than four	2.9 (1.6–5.7)
My preferred position for delivery is lying on the back (supine)	5.9 (3.5–11.1)
The decision to seek antenatal care is generally made by myself	1.9 (1.1–3.4)
Having a complication during immediate past delivery	4.2 (3.0–8.2)

## Discussion

The current study adds value to previous studies of predictors of health facility delivery on two fronts. First since physical distance is a know barrier to delivery in health units only women that have good access to health facilities with delivery services (within 5 km of a maternity facility) are included. This inclusion helps to identify factors that are still important predictors of health facility delivery when physical distance is no longer a barrier [15]. Second, this study tests the significance of beliefs identified in previous qualitative research [16-18] in influencing choice of delivery site within the Ugandan setting. Data from the qualitative studies helped us to design a questionnaire for the current quantitative aspect.

Furthermore, the previous qualitative studies helps us to understand why some of the independent predictors identified in this study such as disposal of the placenta and delivery position are probably important in influencing the place of delivery [16-18]. Women were reported to be shy about exposing their genitals during child birth and hence preferred squatting or kneeling with people they are not used to [17,18]. Because these alternative delivery positions during delivery are not offered in health facilities in Uganda some women preferred to deliver in the community. Besides, the alternative delivery positions (such as squatting or kneeling) are believed by women in the Ugandan setting to make labour easy and fast. Indeed, there is concurrence with this belief in published literature in many parts the world [19-23].

In Uganda there is a strong belief that the placenta is the “second child” and is therefore supposed to be handled with care and disposed off properly. The disposal of the placental involves many traditional rituals as it is associated with luck, misfortunes, survival of the child and also determines whether the mother delivers without any complications during subsequent pregnancies [17,18]. Thus disposal of the placenta being an independent predictor of place of delivery could be linked to these strong beliefs.

The independent predictors of health facility delivery identified in this study can be classified into three groups. First are variables that are useful in identifying mothers who are less likely to deliver in health facilities such as those from households of low social economic status and those mothers who have at least 4 births. These mothers require intense health education regarding the benefits of health facility deliveries. Furthermore, mothers from low socio-economic households require more support regarding income generating activities that improve household incomes. Experience from Bangladesh has shown that women who are supported with loans from micro-credit programmes to start small income generating activities improve their household incomes [24] and consequently their health care seeking behaviour including supervised institutional delivery [25].

Second are variables that identify barriers or supports (such as access to transport and autonomy in decision making) to enable women to deliver in health facilities. Whereas access to transport in improving delivery at health facilities is expected and easily explained, there was a paradox regarding autonomy in decision making. Women who made the decision to attend antenatal on their own were more likely to deliver in health facilities but women who had autonomy in deciding on place of delivery were less likely to deliver in health facilities. A possible explanation for this paradox is that women are more vulnerable during labour compared to during the antenatal period. That is to say that a woman is more likely to require the assistance of other people during labour compared to during antenatal period. Such help may include help with things like organizing transport, accompanying women to place of delivery, attending to physical, financial and emotional needs as well as with care of the newborn. The implication of this finding is that other people particularly the spouse should be part and partial of birth plan. Indeed spouse involvement is a known strategy for improving reproductive health outcomes [26-28] including supervised institutional deliveries [29].

Third are salient beliefs that may influence delivery within the health facility (such as regarding the disposal of placenta and delivery position) and previous delivery experience (such as having a complication or not). The role of beliefs, and previous delivery experience in influencing place of delivery would be expected and could also be postulated from applied social psychology models and theory of health education/ promotion [30,31]. The implications of these findings for improving the proportion of women who deliver in health units are multiple. First there is need to consider the use of alternative delivery positions with women being allowed to adopt the position they find most comfortable in health facilities in Uganda as already done in other countries [32]. Second people who supervise deliveries also need the appropriate skills to manage labour in the alternative delivery positions. Third the data also suggest that the health education given to mothers and their significant others should stress that the occurrence of a complication is difficult to predict and can occur unexpectedly and during any pregnancy. Finally, supervisors of deliveries in health facilities may ask the women or their attendants regarding their preferred mode of disposal of the placenta. People who choose to dispose of the placenta themselves may be instructed in safe carriage and disposal of the placenta.

One limitation of the study was the cross-sectional design making it difficult to establish cause and effect. For example it cannot be said with certainty whether it is the non preference of supine position or not trusting health workers in disposal of placenta that inhibit health facility delivery or whether mothers with these beliefs are more likely to deliver at home. A second weakness was the use of self report regarding place of delivery and on other study variables which could not be verified by reliable records. However, previous reports [9] have found verbal interviews especially within the past five years to be a reliable way of assessing women’s place of delivery. Besides events surrounding child birth are important events in a woman’s life and are therefore less likely to be forgotten. Furthermore, the same data were used to perform a large number of statistical tests with the same outcome variable. As these tests may not be statistically independent, there is increased likelihood of finding spurious significant results.

## Conclusions

Inspite of these limitations, this study contributes to the knowledge about determinants for health facility delivery. Since most of the predictors for health facility deliveries are now know [11-17], future research should test interventions that increase deliveries in health units [25-29].

## Competing interests

The authors declare that they have no competing interests.

## Authors' contributions

AA: participated in conception, design and acquisition of the data. She analysed and interpreted the data and has given final approval of version to be published. DM: participated in interpretation of data, been involved in revising the manuscript and given final approval of version to be published. GBO: contributed to conception and acquisition of data, and given final approval of version to be published. FN: made contributions to conception, design, analysis and interpretation of data; lead the drafting of the manuscript and given final approval of version to be published.

## Authors’ information

AA has recently completed training for masters in Public health. She previously worked as medical superintendent of St. Anthony’s hospital in Tororo, Uganda.

DM Heads the African epidemiology network and is pursuing studies leading to the award of a PhD at Karolinska Instiutet, Stockholm, Sweden.

GBO is the District health officer for Busia district in Uganda with wide experience in health service delivery and evaluation in rural areas.

FN is a teacher of public health at Makerere University. Research interests include determinants and interventions to deliver health services.

## Pre-publication history

The pre-publication history for this paper can be accessed here:

http://www.biomedcentral.com/1471-2393/12/132/prepub
